# An ethical framework adapted for infection prevention and control

**DOI:** 10.1017/ice.2023.121

**Published:** 2023-12

**Authors:** Charlie Tan, Marianna Ofner, Heather L. Candon, Kevin Reel, Sally Bean, Adrienne K. Chan, Jerome A. Leis

**Affiliations:** 1 Infection Prevention and Control, Sunnybrook Health Sciences Centre, Toronto, Ontario, Canada; 2 Division of Infectious Diseases, Department of Medicine, University of Toronto, Toronto, Ontario, Canada; 3 Dalla Lana School of Public Health, University of Toronto, Toronto, Ontario, Canada; 4 Joint Centre for Bioethics, University of Toronto, Toronto, Ontario, Canada; 5 Department of Occupational Science and Occupational Therapy, University of Toronto, Ontario, Canada

## Abstract

**Objective:**

The ethical implications of infection prevention and control (IPAC) are recognized, yet a framework to guide the application of ethical principles is lacking. We adapted an ethical framework to provide a systematic approach for fair and transparent IPAC decision making.

**Methods:**

We conducted a literature search for existing ethical frameworks in IPAC. Working with practicing healthcare ethicists, an existing ethical framework was adapted for use in IPAC. Indications were developed for application to practice, with integration of ethical principles and process conditions specifically relevant to IPAC. Practical refinements were made to the framework based on end-user feedback and application to 2 real-world situations.

**Results:**

In total, 7 articles were identified that discussed ethical principles within IPAC, but none proposed a systematic framework to guide ethical decision making. The adapted framework, named the Ethical Infection Prevention and Control (EIPAC) framework, takes the user through 4 intuitive and actionable steps, centering key ethical principles that facilitate reasoned and just decision making. In applying the EIPAC framework to practice, weighing the predefined ethical principles in different scenarios was a challenge. Although no hierarchy of principles can apply to all contexts in IPAC, our experience highlighted that the equitable distribution of benefits and burdens, and the proportional impacts of options under review, are particularly important considerations for IPAC.

**Conclusions:**

The EIPAC framework can serve as an actionable ethical principles-based decision-making tool for use by IPAC professionals encountering complex situations in any healthcare context.

Ethical practice is a core tenet of health care, and it is well integrated in clinical medicine, medical research, and public health. Although the ethical implications of infection prevention and control (IPAC) have been described,^1–3^ a framework to guide the application of ethical principles in this field is lacking.

This gap exists despite the frequency with which ethical questions are encountered in IPAC. The decisions that IPAC professionals are faced with are characteristically complex. They involve the considerations not only of individual patients but also of healthcare workers and their institutions, each with distinct viewpoints and priorities. Similar to clinical medicine, recommendations are made based on the best available evidence, whether derived from existing published literature, ongoing surveillance, or iterative quality assessments. However, unlike clinical medicine, in which the autonomy and well-being of the individual patient is generally prioritized, IPAC also applies a public health perspective that includes consideration of overall welfare; justice; and fairness across groups, populations and health systems.^3^ IPAC decisions also frequently involve allocation of finite resources within the pragmatic context of organizational constraints. Such decisions can be incongruent with IPAC best practices, which can lead to ambiguity around the most ethically appropriate course of action. The COVID-19 pandemic has highlighted the need for a more systematic approach for situations when infection control professionals are grappling with complicated questions that have ethical implications, especially in context of considerable evidentiary uncertainty.^4,5^

Ethical frameworks are tools to work through complex ethical questions and establish the most appropriate courses of action using the information available. Given the complex decisions IPAC professionals confront, balancing contrasting fiduciary duties, an ethical framework for IPAC is warranted. We developed an ethical framework specifically adapted for use by IPAC professionals.

## Methods

### Study setting

Sunnybrook Health Sciences Centre is an academic hospital in Toronto, Canada, composed of acute-care, long-term care and rehabilitation facilities, and also supporting long-term care homes, retirement homes and other congregate living facilities in north Toronto since early in the COVID-19 pandemic.^6^ In response to new provincial legislation for long-term care homes in Ontario, Canada in April 2022,^7^ we set out to adapt an ethical framework to guide IPAC decision making across different healthcare settings.

### Literature review

We conducted a literature search for ethical frameworks in IPAC. We searched MEDLINE from database inception to February 3, 2023, using the following search terms: ethics AND (“infection prevention and control” OR “infection prevention” OR “infection control”). Among 1,574 articles, we identified 7 that discussed ethical aspects of IPAC.^1–3,8–11^ The ethical implications of specific topics, such as visitation restrictions,^12^ IPAC in long-term care^13,14^ and surveillance and/or management of antimicrobial-resistant organisms,^15,16^ have also been presented. However, we did not identify any published examples of actionable tools that provide a systematic framework to guide ethical decision making.

### Creation of ethical infection prevention and control framework

We partnered with practicing healthcare ethicists who recommended adapting the IDEA framework^17^ for use in IPAC. This existing framework was created by a network of organizations promoting ethical practice in community care settings,^18^ and it has been used broadly by health and community care organizations in Canada and globally. Adaptations to the IDEA framework have integrated it with the Accountability for Reasonableness framework to explicitly embed both procedural and substantive ethical principles,^17,19,20^ as well as process-based conditions that can improve the acceptability of the decision-making endeavour to affected stakeholders. The IDEA framework is considered easily understandable, intuitive, and readily adaptable to serve decision makers in multiple contexts.

We originally adapted the IDEA framework for IPAC with integration of ethical principles based on provincial legal requirements. It was modified to make it as straightforward and cogent as possible, appropriate for IPAC professionals without existing expertise in medical ethics. The adapted framework, named the Ethical Infection Prevention and Control (EIPAC) framework, was shared among partner facilities, and alterations were made based on end-user experiential feedback. The version presented here departs from specific provincial requirements to include principles that are intended to be more intuitive and less abstract, and that are applicable to various healthcare settings.

### Application to practice

The EIPAC framework was applied to 2 real-world contemporaneous situations that arose in our IPAC program. Some details have been altered to maintain anonymity of those involved. These real case-study examples allowed us to make practical refinements and to gain experience regarding how to weigh the relevant ethical principles.

## Results

### Overview of the EIPAC framework

**Figure 1.**
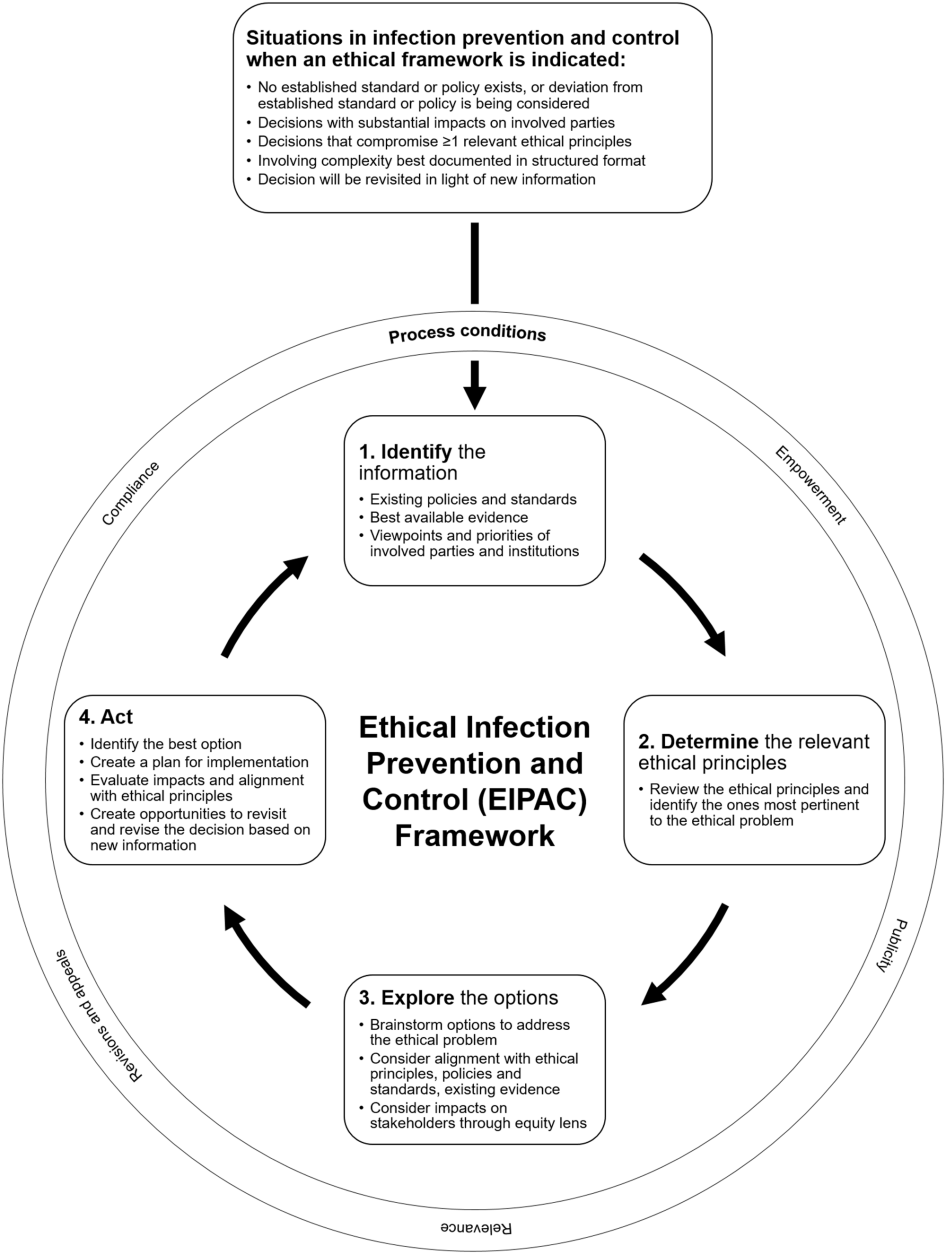
The Ethical Infection Prevention and Control (EIPAC) framework.

The EIPAC framework is summarized in Figure 1. It is depicted as a cycle to emphasize that ethical decision making is dynamic rather than simply linear and that decisions should be re-evaluated as new information emerges. An accompanying worksheet was created to facilitate documentation of the decision-making process, such as when decisions may need to be revisited in the future (Supplementary Material online). The EIPAC framework comprises 4 steps:

1. **Identify the information**, in which the ethical problem is clarified and relevant considerations are ascertained. This includes IPAC policies and best practices, existing regulations and standards, affected stakeholders and their viewpoints and priorities, and best available evidence.
2. **Determine the relevant ethical principles**, in which ethical principles specific to IPAC are considered and germane principles are brought forward for deliberation (Box 1). This step requires an examination of how the principles apply to the specific situation, as well as a judgment about the centrality of some principles over others. Those involved in decision making should deliberate upon the tensions between principles, and determine which principles ought to carry more weight, and why.
3. **Explore the options**, in which solutions for the ethical problem are brainstormed, with consideration of alignment with the evidence, policies, and regulations identified in Step 1 and the ethical principles identified in Step 2. Impacts should be assessed through an equity lens, recognizing the different circumstances and needs of affected parties. An equity lens consciously evaluates how benefits and burdens are distributed across parties and seeks to minimize existing disparities, often linked to the effects of the social determinants of health.^21^ Equity is specifically included as an ethical principle to center it during the decision-making process (Box 1).
4. **Act**, in which the most ethically sound option is identified, a plan is created for implementation, and mechanisms to evaluate the impacts of the decision are ensured.

Box 1. Ethical Principles Relevant to Infection Prevention and Control

- **Autonomy** – Respect people’s right to self-determination and ability to make informed decisions regarding their care.
- **Beneficence** – Decisions should strive to improve the welfare and well-being of affected parties.
- **Equity and justice** – Benefits and possible harms associated with a decision should be distributed between affected parties in a fair and balanced manner, being mindful of existing health disparities and taking care not to exacerbate them. These disparities can be due to systematic and structural inequities among groups of people related to social, economic, demographic or geographic factors, or by other dimensions of inequality (eg, gender identification, racialization, disability, or sexual orientation).^21^
- **Evidence** – Consider the best available evidence, relevant infection prevention and control standards or policies, and best practices based on prior experiences.
- **Nonmaleficence** – Avoid decisions that will or are at high risk to cause harm to individuals.
- **Proportionality** – The potential impacts of options being considered for implementation should be commensurate to the associated level of risk if those options are not taken.
- **Reciprocity** – Endeavour to mitigate the burdens imposed on affected parties as much as possible.
- **Transparency** – Endeavour to make the decision-making process clear and open to involved stakeholders.

In addition to these 4 steps, the EIPAC framework includes 5 process-based conditions that ensure that the decision-making process is viewed as fair and transparent by all involved parties (Box 2). The conditions help to actively involve relevant stakeholders in the decision-making process and take account of their perspectives, to prioritize transparency and accountability to all parties, and to create mechanisms to re-evaluate decisions as new information emerges.

Box 2. Process Conditions for Ethical Decision Making

- **Empowerment** – Include all affected parties in the decision-making process as much as possible, with efforts to minimize power differences and optimize effective opportunities for participation.
- **Publicity** – Ensure that the decision-making process is transparent and accessible to relevant stakeholders.
- **Relevance** – Decisions should be made based on reasons (eg, best available evidence, ethical principles, standards and policies) that “fair-minded” people can agree are relevant under the circumstances.
- **Revisions and appeals** – Create opportunities to revisit and revise decisions when new evidence or considerations arise, as well as mechanisms to challenge decisions and resolve disputes.
- **Compliance** – Prioritize accountability, ensuring that the decision-making process is in alignment with the 4 other process conditions.

### Application of the EIPAC framework—Scenario 1

A long-term care home is in COVID-19 outbreak. In one of the semiprivate rooms housing 2 residents on the outbreak unit, one of the residents (ie, the case resident) tests positive for COVID-19, while the other resident (ie, the roommate) tests negative. No single-resident isolation rooms are available at this facility.

1. **Identify the information:** Institutional COVID-19 policy and regional best-practice recommendations advise placing residents with COVID-19 in single rooms under transmission-based precautions.^22,23^ Multiple studies demonstrate high secondary attack rates among roommates of individuals with COVID-19 infection.^24,25^ Affected stakeholders included the roommate, who prioritized reducing their personal risk of acquiring COVID-19. The case resident prioritized staying in their own room, which was their home and where they were most comfortable. Other noninfected residents in the facility, including those who the roommate would need to room with to separate from the case resident, prioritized reducing their individual risks of COVID-19 infection. The staff in the facility prioritized reducing the risk of the roommate acquiring COVID-19, as well as minimizing resident exposures and propagation of spread. The long-term care home prioritized reducing the overall burden of COVID-19 in the facility and optimizing the overall welfare of all residents.
2. **Determine the ethical principles:** The predefined ethical principles were considered. Principles felt to be most germane included autonomy, proportionality, equity and justice, and transparency (Box 1). While always relevant, beneficence, nonmaleficence, and evidence were considered more neutral dimensions in this instance and were not expected to sway the balance of possible options.
3. **Explore the options:** Three options were considered. Option 1 was moving the roommate to a different semiprivate room on the same unit, which would have been shared with another resident who did not have COVID-19. Option 2 was moving the roommate to a semiprivate room on a separate nonoutbreak unit where no residents had COVID-19. Option 3 was keeping the roommate in the current room with the case resident. The best available evidence supports separation of contacts from cases, which may not be possible in situations of resource constraints.^22,23^ Although options 1 and 2 would achieve this, when considering the ethical principles of equity and justice, they both conferred burdens to other residents in the form of risk of SARS-CoV-2 transmission, either to a nonexposed roommate (option 1) or to a nonoutbreak unit (option 2). These burdens were viewed through an equity lens, recognizing that many residents in the facility had comorbidities and disabilities that made them vulnerable to poor outcomes from COVID-19 infection. Option 3 had the benefit of minimizing burdens to other residents, staff, and the facility, but ongoing exposure of the case resident to the roommate would need to be mitigated as much as possible. Regarding the principle of proportionality, there were concerns that the risks mitigated by options 1 and 2 (separation of the roommate from the case resident) were not commensurate with the associated risks of possibly exposing a nonexposed resident (option 1) or a nonoutbreak unit (option 2). Lastly, transparency was prioritized by involving the long-term care home staff and leadership in decision making, as well as explaining the situation to the affected residents.
4. **Act:** Option 3 was selected as the best option because the roommate had already been exposed to the case resident and because options 1 and 2 would have disproportionately conferred burdens to other residents in the facility. Mitigation measures offered to the roommate included masking of both the roommate and case resident, opening windows, closing the curtain between the beds, and providing a dedicated washroom for the case resident. The process was documented to allow the decision to be revisited in the future, if needed. The decision was prospectively evaluated via regular symptom monitoring and a postexposure surveillance SARS-CoV-2 test for the roommate.

Throughout application of the EIPAC framework, the process-based conditions were prioritized at all steps to ensure the decision-making process was fair and transparent (Box 2). This included actively engaging residents and their families, frontline staff, and long-term care home leadership; documenting the decision-making process; and making records accessible for review by all parties.

### Application of the EIPAC framework—Scenario 2

A celebration of life was organized for a resident (“Mr. A”) with terminal illness who was scheduled for medical assistance in dying (MAiD)^26^ the following day. He had invited several other residents, including individuals living on other units, as well as friends and family living in the community. A catered family-style dinner had been planned. The week of the celebration, a norovirus outbreak was declared on Mr. A’s unit. Mr. A was asymptomatic and intended to proceed with MAiD; he requested that the celebration of life take place as scheduled.

1. **Identify the information:** Guidelines and regional best practice recommendations advise restricting nonessential visitors and events involving congregation during gastroenteritis outbreaks.^27,28^ Norovirus is highly communicable, with a low infectious dose required to cause gastroenteritis,^29^ and studies have demonstrated high secondary attack rates during outbreaks in various settings, including in congregate living facilities.^30,31^ The celebration of life could not be rescheduled because Mr. A’s MAiD procedure had been arranged for the following day, and he wished to proceed as planned. Impacted stakeholders included Mr. A, who prioritized holding this last opportunity to see his family and friends. Residents invited to the celebration, as well as community-dwelling guests, prioritized attending the celebration with Mr. A. Other residents in the facility prioritized reducing their individual risks of norovirus infection. The staff and facility leadership prioritized reducing propagation of the outbreak to additional residents throughout the home but also recognized the importance of respecting Mr. A’s wishes to hold his celebration of life.
2. **Determine the ethical principles:** The ethical principles felt to be most germane included autonomy, beneficence, nonmaleficence, proportionality, and reciprocity (Box 1). In this situation, autonomy was considered central throughout Mr. A’s consideration of and eventual plan for MAiD. Preserving that plan and the benefits he, and others, would derive from it were essential features of the ethical issue. These benefits were clearly inconsistent with potential harms of disrupting this plan, hence the importance of nonmaleficence. Given the possibility that infection control measures could restrict Mr. A’s end-of-life arrangements, proportionality and reciprocity were important considerations as well.
3. **Explore the options:** Three options were considered. Option 1 was cancelling the celebration of life altogether in the setting of norovirus outbreak. Option 2 was proceeding with the celebration of life but with only community-dwelling guests invited (ie, no facility-dwelling guests). Option 3 was holding the celebration of life as scheduled with all invited guests in attendance. Option 1 was best aligned with existing policies and best practices for norovirus outbreaks. By reducing opportunities for forward transmission, it also prioritized beneficence and nonmaleficence for other residents in the facility. However, when viewed through the ethical principle of proportionality, option 1 conferred substantial burdens to Mr. A, as well as invited friends and family, by depriving them of their last opportunity to celebrate Mr. A’s life. Option 2 conferred fewer burdens to Mr. A by allowing community-dwelling guests to attend, who would pose a lower risk of norovirus propagation in the home compared to residents. However, the presence of fellow residents was particularly meaningful to Mr. A given their shared daily life for several years. Lastly, option 3 placed the greatest priority on Mr. A’s individual interests but with higher associated risk of norovirus transmission throughout the facility.
4. **Act:** Because Mr. A wished to continue with his selected date for MAiD, his autonomy and individual interests, and acting in a beneficent manner toward him, were prioritized heavily in decision making. The burdens imposed by options 1 and 2 on Mr. A were felt to be disproportionate to the associated risk of norovirus transmission. Option 3 was aligned with the ethical principle of proportionality as long as appropriate IPAC mitigations were in place. These included education and screening of guests for gastrointestinal symptoms, exclusion of symptomatic residents from attending unless recovered for at least 48 hours, making alcohol-based hand rub widely available and encouraging frequent hand hygiene, serving facility-residing guests separately such that they did not participate in family-style dining, and disinfecting the event space with a product active against nonenveloped viruses. Heightened surveillance for gastrointestinal illness was implemented among the residents who attended the event with low threshold for instituting contact precautions.

As in scenario 1, the process-based conditions were emphasized throughout the decision-making process (Box 2). The decision was reviewed with the long-term care home leadership who agreed that the factors considered were relevant and were prioritized in a reasonable manner. The decision and required measures were communicated to Mr. A and his guests in advance of the celebration, and they were agreeable to abiding by these requirements. Syndromic follow-up was used to identify new symptomatic cases among those who attended the celebration of life.

## Discussion

IPAC decisions are made every day that involve principles, beliefs, and values. Most decision making is well served by informal deliberation using these guideposts. However, in complex situations, a structured approach can facilitate principled decision making that is as fair and transparent as possible.

Although numerous ethical frameworks for patient care and public health exist, it has long been recognized that IPAC presents differences that warrant its own specific framework.^3^ Public health focuses on health protection at the overall population level, with large segments that are healthy and sporadically access the healthcare system. Although similar in its goal, IPAC is centered on hospitalized and institutionalized patients who are more vulnerable to infections and have limited independent recourse to alter their risk. The EIPAC framework is an adapted decision-making tool that includes ethical principles most relevant to this context (Box 1).

In our application of the EIPAC framework, one challenge has been determining how best to weigh ethical principles in selecting the most appropriate option. Specifying, prioritizing, and balancing ethical principles is a perennial difficulty when applying a principles-based framework, and no hierarchy of principles can apply to all contexts.^32^ Because different problems in IPAC have unique situational contexts, it is difficult to be broadly prescriptive regarding how principles should be balanced. In our experience, the equitable distribution of benefits and burdens and the proportional impacts of options under review are particularly important considerations for IPAC. In the first scenario, our IPAC team felt that the burdens conferred by separating the roommate from the case resident would be inequitably distributed to other residents in the facility. In the second example, the principle of proportionality required our IPAC team to assess whether the burdens of infection risk were truly commensurate with the burdens imposed by conventional control measures, which would deprive a resident from their last opportunity to gather with their friends and family. We also acknowledge that ethical principles may be weighed differently by different practitioners, but embedding fair process conditions within the EIPAC framework helps to ensure that procedural justice is supported and the ethical principles are balanced in a manner viewed as fair and transparent.

The EIPAC framework has several limitations. It was originally developed to respond to our local context and provincial requirements and does not necessarily reflect existing legal or regulatory expectations in other jurisdictions. However, the ethical principles and structure of this framework can be applied broadly, and IPAC programs can adapt it for their specific needs. It may be challenging for IPAC professionals without expertise in medical ethics to identify and prioritize the ethical principles, as well as to balance considerations of their institutions and health systems. Through iterative applications of the framework and its principles, IPAC professionals may become increasingly comfortable with ethics-based decision making. Collaborating with practicing healthcare ethicists is also encouraged.

In summary, an ethical framework can provide a systematic approach for fair and transparent decision making in IPAC. The EIPAC framework can serve as an actionable ethical principles-based decision-making tool for use by IPAC professionals encountering complex situations in any healthcare context.

## Supplementary material

For supplementary material accompanying this paper visit https://doi.org/10.1017/ice.2023.121. click here to view supplementary material
