# Supplementary material for: An ethical framework adapted for infection prevention and control
Source: Infect Control Hosp Epidemiol. 2023 Jul 10;44(12):2044–9. doi: 10.1017/ice.2023.121 (PMC10755160; doi:10.1017/ice.2023.121)
Supplement: Supplementary file 1 [file S0899823X23001216sup001.docx]

**Supplementary Material**

**Ethical Infection Prevention and Control (EIPAC) framework worksheet**

| **Step 1: Identify** the information |
| --- |
| *What is the presenting ethical problem?* |
| *What are the relevant infection prevention and control policies and best practices (such as those specific to your institution or local context)? This can include formal policies as well as practices derived from previous experiences with similar ethical problems.* |
| *What are the existing regulations and standards that need to be followed?* |
| *Who are the impacted stakeholders (such as residents, families, staff, institutions, health systems) and what are their viewpoints and priorities?* |
| *What is the best available evidence?* |

| **Step 2: Determine** the relevant ethical principles |
| --- |
| *Review the following ethical principles and ask yourself the associated questions. Determine which principles are most relevant to the ethical problem.*   - **Autonomy** – Do affected parties have the opportunity to make informed decisions regarding their own care? - **Beneficence** – Is the welfare and wellbeing of affected parties being prioritized? - **Equity and justice** – How are the impacts of the decision distributed among affected parties, and how are existing health disparities addressed? - **Evidence** – Is there existing evidence relevant to this ethical problem (identified in Step 1), and how can it inform decision-making? - **Non-maleficence** – What are the possible burdens and harms imposed on affected parties, and how can these be avoided? - **Proportionality** – Are the potential effects of decisions being considered to address the ethical problem commensurate with the level of risk associated with the problem? - **Reciprocity** – Are there mitigation measures that can be proposed to minimize the burdens of the decision on affected parties? - **Transparency** – Is the deliberation process clear and open to affected parties? |
| *Are there any other factors that need to be considered?* |
|  |

| **Step 3: Explore** the options | | |
| --- | --- | --- |
| *Option 1:* | *Option 2:* | *Option 3:* |
| Alignment with IPAC policies and best practices (from Step 1) | Alignment with IPAC policies and best practices (from Step 1) | Alignment with IPAC policies and best practices (from Step 1) |
| Alignment with existing regulations and standards (from Step 1) | Alignment with existing regulations and standards (from Step 1) | Alignment with existing regulations and standards (from Step 1) |
| Alignment with ethical principles (from Step 2) | Alignment with ethical principles (from Step 2) | Alignment with ethical principles (from Step 2) |
| What are the possible impacts and how are they distributed among affected parties? | What are the possible impacts and how are they distributed among affected parties? | What are the possible impacts and how are they distributed among affected parties? |
| **What is the most ethically justifiable option?** | | |
|  | | |

| **Step 4: Act** | | |
| --- | --- | --- |
| *Documentation of decision:* | | |
| *Implementation plan:* | | |
| *Evaluation plan:* | | |
| **Reviewed by:** | **Date:** | **Time:** |
